# Iron Status, Comparators, and the Interpretation of Red Cell Distribution Width in Inflammatory Bowel Disease

**DOI:** 10.5152/tjg.2026.26515

**Published:** 2026-09-07

**Authors:** Remzi Ekici

**Affiliations:** 1 Department of Gastroenterology, Gülhane Training and Research Hospital, University of Health Sciences, Ankara, Türkiye

To the Editor,

Özden reports that red cell distribution width (RDW) is independently associated with objective inflammatory activity in inflammatory bowel disease (IBD) and provides an incremental value during treat-to-target monitoring.^1^ The large cohort, stringent definitions of endoscopic and histologic outcomes, and optimism-corrected estimates are commendable. However, we believe that 2methodological issues warrant clarification before these conclusions are translated into clinical practice.

First, the Abstract states that the multivariable analyses were adjusted for anemia, iron-related parameters, inflammatory markers, disease extent, and medications, and the Introduction identifies inadequate adjustment for iron-related parameters as a key limitation of previous studies. However, Supplementary Table 3 in the article includes hemoglobin and mean corpuscular volume, but neither ferritin nor transferrin saturation. Hemoglobin levels help identify anemia, and mean corpuscular volume reflects erythrocyte size; neither directly reflects iron availability. Both ferritin and transferrin saturation were measured and were significantly lower in active disease (65 vs 85 ng/mL, *P* = .021; 18% vs 24%, *P* = .012). Both were also inversely correlated with RDW (*r* = −0.25 and −0.29; *P* < .001), suggesting that they remain plausible sources of residual confounding because they were not included in the adjusted models. Excluding patients with ferritin concentrations below 30 ng/mL does not fully address this concern because, according to ECCO recommendations, ferritin concentrations below 100 µg/L may still indicate iron deficiency in the presence of inflammation.^2^ Accordingly, excluding patients with ferritin concentrations below 30 ng/mL cannot be assumed to eliminate iron deficiency in an inflamed IBD cohort; indeed, the median ferritin concentration in the active disease group was 65 ng/mL (interquartile range: 30-120). Likewise, immunomodulator use was not included in the adjusted model despite its potential effects on erythrocyte indices.

Second, statistical increment should not be equated with clinical increment. Adding RDW to C-reactive protein increaimprove language and edit grammarsed the area under the curve from 0.845 to 0.880; however, fecal calprotectin alone achieved an area under the curve of 0.875 in the same cohort. RDW was neither directly compared with fecal calprotectin nor evaluated for incremental value beyond it, and measures of clinical utility, such as decision curve analysis or net reclassification, were not reported.

These findings support an association between RDW and inflammatory activity. However, claims of iron-independent and clinically incremental utility would require models incorporating the measured iron-status variables and validation against clinically relevant comparators. Until then, the proposed 14.5% threshold should be interpreted cautiously before being applied to routine IBD practice, where iron deficiency remains common.

## Data Availability

The author used ChatGPT (OpenAI) and claude solely to improve language and edit grammar during the preparation of the manuscript. The author reviewed and approved the final text and takes full responsibility for the content
